# *Mycobacterium leprae* genomes from a British medieval leprosy hospital: towards understanding an ancient epidemic

**DOI:** 10.1186/1471-2164-15-270

**Published:** 2014-04-08

**Authors:** Tom A Mendum, Verena J Schuenemann, Simon Roffey, G Michael Taylor, Huihai Wu, Pushpendra Singh, Katie Tucker, Jason Hinds, Stewart T Cole, Andrzej M Kierzek, Kay Nieselt, Johannes Krause, Graham R Stewart

**Affiliations:** 1 Department of Microbial and Cellular Sciences, Faculty of Health and Medical Sciences, University of Surrey, GU2 7XH Guildford, UK; 2 Institute for Archaeological Sciences, University of Tübingen, Rümelinstr 23, 72070 Tübingen, Germany; 3 Department of Archaeology, University of Winchester, Winchester, UK; 4 Global Health Institute, École Polytechnique Fédérale de Lausanne, Lausanne, Switzerland; 5 Bacterial Microarray Group, Division of Cellular and Molecular Medicine, St. George’s, University of London, Cranmer Terrace, London, UK; 6 Center for Bioinformatics, University of Tübingen, 72076 Tübingen, Germany

**Keywords:** *Mycobacterium leprae*, SNP, Leprosy, Genome, Ancient DNA, Middle Ages, Bioarchaeology

## Abstract

**Background:**

Leprosy has afflicted humankind throughout history leaving evidence in both early texts and the archaeological record. In Britain, leprosy was widespread throughout the Middle Ages until its gradual and unexplained decline between the 14^th^ and 16^th^ centuries. The nature of this ancient endemic leprosy and its relationship to modern strains is only partly understood. Modern leprosy strains are currently divided into 5 phylogenetic groups, types 0 to 4, each with strong geographical links. Until recently, European strains, both ancient and modern, were thought to be exclusively type 3 strains. However, evidence for type 2 strains, a group normally associated with Central Asia and the Middle East, has recently been found in archaeological samples in Scandinavia and from two skeletons from the medieval leprosy hospital (or *leprosarium*) of St Mary Magdalen, near Winchester, England.

**Results:**

Here we report the genotypic analysis and whole genome sequencing of two further ancient *M. leprae* genomes extracted from the remains of two individuals, Sk14 and Sk27, that were excavated from 10^th^-12^th^ century burials at the *leprosarium* of St Mary Magdalen. DNA was extracted from the surfaces of bones showing osteological signs of leprosy. Known *M. leprae* polymorphisms were PCR amplified and Sanger sequenced, while draft genomes were generated by enriching for *M. leprae* DNA, and Illumina sequencing. SNP-typing and phylogenetic analysis of the draft genomes placed both of these ancient strains in the conserved type 2 group, with very few novel SNPs compared to other ancient or modern strains.

**Conclusions:**

The genomes of the two newly sequenced *M. leprae* strains group firmly with other type 2F strains. Moreover, the *M. leprae* strain most closely related to one of the strains, Sk14, in the worldwide phylogeny is a contemporaneous ancient St Magdalen skeleton, vividly illustrating the epidemic and clonal nature of leprosy at this site. The prevalence of these type 2 strains indicates that type 2F strains, in contrast to later European and associated North American type 3 isolates, may have been the co-dominant or even the predominant genotype at this location during the 11^th^ century.

## Background

Leprosy has been known since the earliest recorded times, with references in ancient texts [1] and paleopathological evidence in the archaeological record dating back at least 4000 years [2]. In Britain, evidence for leprosy has been recorded from as early as the 4^th^ century AD [3] but is thought to have been particularly prevalent between the 11^th^ and 14^th^ centuries, as evidenced by a rise in the number of active leprosy hospitals known as *leprosaria*[4]. However, by the end of this period, leprosy was declining such that *leprosaria* were being abandoned or put to other uses. Firstly in southern Britain but later in regions further to the north, until by the 18^th^ century leprosy was only recorded in the far northern isles of Shetland, the last sufferer dying in the Edinburgh Infirmary in 1798 [5]. This decline in the prevalence of leprosy was reflected throughout Europe and remains an intriguing feature of infectious disease history for which there is no clear reason. Although leprosy is now rare in Europe, it remains a significant disease in many parts of the world with approximately 220,000 new cases in 2011 [6]. Despite the use of multi-drug therapy against leprosy since the 1980s, the prevalence of the disease remains stubbornly high in many areas. A wider understanding of the origins and history of leprosy, including why leprosy died out in Europe, may help to develop more effective strategies for controlling the modern disease.

Leprosy genomes show an unusual degree of conservation with strains being >99.9% identical. Only 807 polymorphic sites are recorded across all known strains [7], most of which are Single Nucleotide Polymorphisms (SNPs), with some Variable Nucleotide Tandem Repeats (VNTRs) [8]. This is despite the presence of more than 1300 pseudogenes, equating to 41% of the genome [9]. A subset of these polymorphic loci were used to develop a molecular typing scheme and to generate a phylogeny for *M. leprae*. This predicted an ancestral strain, thought to have a genotype between that of modern type 2 and type 3 strains, originating in East Africa approximately 100,000 years ago [8]. From there, leprosy appeared to have disseminated across the world along routes of human migration and trade, with type 1 strains dominating in Southeast Asia, type 3 s in the Near East and Europe, type 3Is in Northwestern Europe and ultimately in North America and type 4 s in West Africa and later Brazil [8]. Recently, new data have challenged this interpretation, with an apparently more basal group, type 0, being identified in patients from China and New Caledonia [7].

In an effort to build a more comprehensive and accurate history for leprosy that better informs our understanding of its origins and macroecology, we and others have been studying DNA from ancient *M. leprae* infections. Leprosy is unusual in that multibacillary infection causes pathological changes of the bone, such that ancient infections can be retrospectively identified from skeletal remains. Using SNP typing and whole genome sequencing of DNA from such lesions, we have recently been able to re-assess the genetic history of leprosy and demonstrate that 1000 years ago European leprosy was caused by strains that group with type 2 and type 3 strains [7,10] and that a most recent common ancestor of all extant and ancient *M. leprae* existed only 1400 to 2700 years ago [7].

In this study we extend this work to gain further insights into the molecular epidemiology of European medieval leprosy. By sequencing the genomes of two further *M. leprae* strains from individuals interred at the *leprosarium* of St Mary Magdalen, Winchester, England, during the second half of the 11^th^ century we can begin to understand in more detail the epidemiology of *M. leprae* at this site [11].

## Results

### Archaeology and osteology

Excavations at St Mary Magdalen, Winchester, are amongst the most extensive of any British leprosy hospital to date [11,12]. The hospital is one of the earliest such institutions known in Britain and, as such, provides insights into both early institutional care and the nature and status of such communities at a relatively early date. Such hospitals may well have been a model for succeeding charitable institutions of social care. Thirty-eight burials have been excavated from the site, of which 33 (87%) show osteological signs of leprosy, a much higher percentage than observed in other British material. Of these, five *M. leprae* strains have been SNP genotyped (Sk2, Sk7, Sk8, and Sk14, and Sk19) [10] and 2 whole genomes (Sk2 and Sk8) sequenced [7].

The current study focuses on typing a further burial, Sk27, and generating whole genome sequences of the *M. leprae* from both Sk14 and Sk27. Both were excavated from the earlier and more northern of two cemeteries at the site (together with skeletons Sk7, Sk8, Sk9 and Sk19 [10]). Based upon comparative and diagnostic material this cemetery was thought to have been in use primarily in the decades immediately following the Norman Conquest, between 1070 AD and 1100 AD. This interpretation was confirmed by ^14^C dating, with dates of between 940 cal AD and 1160 cal AD [11]. Sk14 and Sk27 were ^14^C dated to between 955–1033 cal AD (WK 28629) and 1020–1162 cal AD (SUERC-39676), respectively.

**Figure 1:**
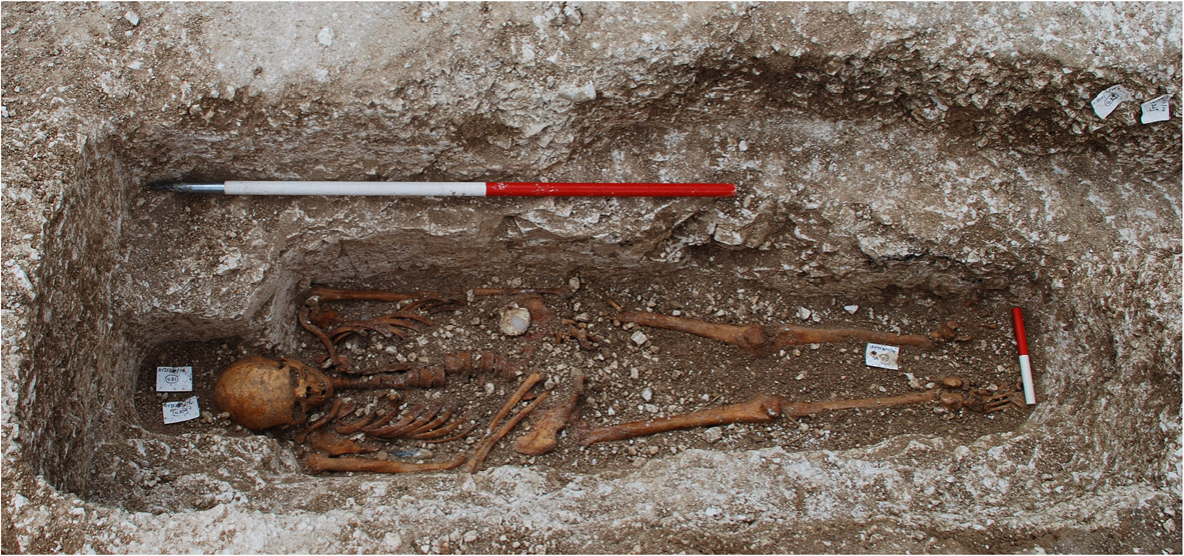
The burial of Sk27 showing the associated scallop shell.

The remains of Sk14 are those of an adolescent, probably male, displaying clear pathological features of leprosy: circumferential wasting of the foot phalanges, loss of bone of the distal ends of the distal foot phalanges, porosity of the shafts of the metatarsals and porous, woven and compact bone on the shafts of the fibulae, thinning of the palate and nasal bones and rounding of the margins of the nasal aperture [13]. In contrast, Sk27 was a middle-aged adult, with only minimal evidence of characteristic leprosy lesions. Indeed, Sk27 was originally identified as a possible control, non-lepromatous skeleton and only on closer examination was porosity and resorption of bone noted on the dorsal surface of the distal ends of the distal foot phalanges, particularly those for the first metatarsal. The grave of Sk27 was also notable for containing a scallop shell (Figure 1), indicating that the remains are likely those of a pilgrim who had completed the Way of St James to the Cathedral of Santiago de Compostela in Galicia, Spain [11].

### Confirmation that skeleton Sk27 had been infected with *M. leprae*

Samples of bone from Sk27 were scraped from the rhino-maxillary area under clean and controlled conditions using disposable gloves, scalpels and tubes. A control sample was taken from around the vomer (the thin bone that separates the nostrils) of a skeleton, Sk12, which showed no osteological evidence of *M. leprae.* The vomer is a site that often has a high *M. leprae* burden in lepromatous leprosy, and so is a good site from which to isolate *M. leprae* DNA. The presence of *M. leprae* DNA in the Sk14 extract was previously confirmed by real-time PCR using primers for the multicopy RLEP and the single copy locus, ML1795 (encoding the 18 kDa antigen), both of which gave single bands of the expected size (111 bp and 114 bp, respectively) [13]. The presence of *M. leprae* DNA in extracts from SK27 was similarly confirmed by PCR. PCR for the multicopy *M. tuberculosis* loci IS*1081* gave no products (79 bp expected) for either sample, indicating an absence of tuberculosis. The control sample Sk12 gave no products with primers for loci of either *M. leprae* or *M. tuberculosis*, indicating that contamination of the samples from extraneous sources or cross-contamination between the samples was negligible.

### Whole genome sequencing of *M. leprae* genomes from skeletons SK14 and Sk27

DNA from skeletons Sk14 and Sk27 was amplified, enriched for *M. leprae* sequences using microarrays, and sequenced as described previously for Sk2 and Sk8 [7]. Details of the read depths, their alignment to the *M. leprae* TN reference genome and percentage genome coverage are given in Table 1. After quality controls less than 1% of the sequence reads aligned with human DNA, even in the control sample Sk12, only 1.8% of reads aligned to human DNA.

**Table 1:** Details of the skeletons excavated from St Mary Magdalen, Winchester

**Skeleton**	**Leprosy-like pathology**	**Fold coverage (depth)**	**Percent coverage (>4 reads)**	^**14**^**C date (cal AD, 95% probability)**
Sk2	Feet, cranium	14.9	92.9	1268-1283
Sk8	Limbs, cranium	20.0	96.4	1010-1160
Sk12	None	0.39	0.99	nd
Sk14	Limbs, feet, cranium (extensive)	26.6/44.8^*^	98.9/98^*^	955-1033
Sk27	Feet (minimal)	11.9/6.7^*^	80.0/41.9^*^	1020-1162^*^

Details of the skeletons excavated from St Mary Magdalen, Winchester, and the whole genome sequence analysis of their associated *M. leprae* strains carried out at the University of Surrey and the University of Tübingen, respectively.

*presented in this study, other details were previously published in Schuenemann *et al*., 2013 [7].

The control sample, Sk12, was characterized by very low coverage of the *M. leprae* genome, punctuated by short regions with high coverage and high diversity. This is consistent with a sample containing little or no *M. leprae* sequence, but with some carry-over of DNA from environmental organisms. This again confirms that there was little or no cross-contamination between samples or from modern *M. leprae* DNA during the sample preparation. Many of the reads that were retrieved from Sk12 had similarity to environmental organisms that contain genomic regions that are likely to be conserved across mycobacteriaceae and beyond. Such regions apparently have sufficient similarity to the microarray probes to hybridize, particularly in the absence of competing *M. leprae* DNA, but are sufficiently dissimilar to generate the high diversity observed. Regions with high coverage included approximately 50 genes such as *rpoB*, *tuf*, *fusA* and certain smaller regions within genes that often represent conserved motifs, for example within ABC transporters. Base calls from regions with high coverage in the control sample were not considered reliable and were discounted from the data.

Sanger sequencing of targeted SNPs (Table 2) was in agreement with Illumina sequencing so validating both typing and sequencing methods. Analysis of VNTR genotypes was not possible for either Sk14 or Sk27 as insufficient numbers of reads spanned the ML0058c (21–3) region, while ML2344-ML2345 (AGA)_20_ and ML2172-ML2173 (GTA)_9_ had high coverage in the Sk12, the control sample, and so were deemed unreliable.

**Table 2:** **Genotyping of *M. leprae *** **strains from Sk14 and Sk27 by SNP analysis**

	**SNP loci (nucleotide positions relate to TN genome, pre correction)**								**Indel (copy no.) 17915**
	**14676**	**1642875**	**2935685**	**1133492**	**7614**	**1113923**	**1104235**	**3102787**	
Sk27	C	T	A	T	C	A	C	C	2
Sk14	C	T	A	T	C	A	C	C	2
Type		2				F			

### Analysis of the ancient *M. leprae* genomes

We have previously used PCR and Sanger sequencing of a subset of SNPs [8] to genotype the *M. leprae* from Sk14 as type 2F [10]. Using identical methods, we also identified Sk27 as a type 2F strain (Table 2).

**Figure 2:**
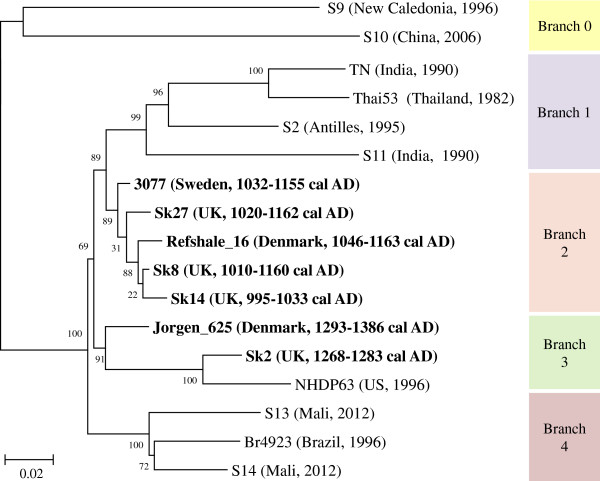
**Phylogenetic relationships between modern and ancient *M. leprae.*** A maximum likelihood tree describing the relationship between Sk14, Sk27 and other ancient (in bold) and modern *M. leprae* genomes [7]. Locations and dates (^14^C estimates or year of isolation) are given in brackets. Groupings are derived from Schuenemann *et al*. [7]. The position of Sk27 is poorly defined but is clearly within group 2 and closely related to other ancient genomes.

Independent analysis of the sequence reads at the University of Tübingen and the University of Surrey (Table 1), generated differing values for coverage and read depth. However, both centres reconstructed very similar genomes with only a single SNP discrepancy (not included in subsequent analysis) between the comparable datasets. Comparison of the Sk14 and Sk27 *M. leprae* genomes with other modern and ancient genomes identified the presence of very few novel polymorphs (Additional file 1: Table S1). Sk14 contained 3 novel indels, all of which were in pseudogenes, and 3 novel SNPs, only one of which was non-synonymous, encoding a change of Gly_163_ to valine in hypothetical protein ML1637. Sk27 had a single novel synonymous SNP and no identifiable novel indels. Phylogenetic analysis generated trees comparable to those of Schuenemann *et al.*[7] and placed Sk14 and Sk27 in the highly conserved type 2F branch of *M. leprae* (Figure 2 and Additional file 2: Figure S1). It is notable that the strain most closely related to Sk14 is another isolate from Winchester, Sk8, described in our previous studies [7,10].

## Discussion

The history of leprosy in Europe is dominated by a drawn-out epidemic that may have lasted a millennium, but peaked between the 11^th^ and 14^th^ centuries before declining during the following centuries. In other parts of the world the incidence of leprosy has remained high despite the administration of multi-drug chemotherapy. Understanding the origins and macroecology of leprosy worldwide, and specifically why it declined in Europe, may help in the development of improved control strategies against the endemic worldwide leprosy of today.

Ancient DNA can be uniquely useful in constructing and assessing models of pathogen evolution [14-16] and macroecological patterns of infection. This is particularly so for *M. leprae*, whose extremely limited genotypic diversity has facilitated the development of models describing the evolution of modern strains and their historic worldwide dissemination. To more fully inform these models and to capture the diversity of *M. leprae* we have begun a bioarchaeology program of research to sequence and reconstruct the genomes of ancient *M. leprae* that infected medieval people at the site of the *leprosarium* of St Mary Magdalen, at Winchester, in southern Britain [7]. Here we continue our characterization of these *M. leprae* strains and present two new 1000 year old mycobacterial genomes, which in combination with previous samples, allow us to describe, in detail, the historical epidemiology of leprosy at this location across the centuries.

Rather than reveal a greater diversity of mycobacterial strains, the genomes that we present are striking in their similarity to modern *M. leprae*, providing further confirmation that the medieval European leprosy epidemic involved strains almost identical to those that today infect people in other parts of the world. It could be argued that this lack of diversity is an artifact of enriching the ancient DNA for *M. leprae* sequence using microarrays based upon modern *M. leprae* genomes, potentially allowing genomic re-arrangements or insertions to go undetected. However, such a scenario would seem unlikely. Firstly, these samples fall within the limits of diversity observed in the *de novo* assembled genomes, both ancient and modern [7-9], none of which have alternative genome architectures. Secondly, we would expect any such changes in genome architecture to be observable in the data as apparent indels at the ends of sequence reads, each representing the boundaries of any such re-arrangement. No such indels were identified in the data. This leaves us to conclude that these ancient strains were indeed highly similar to modern strains and, therefore, the diminishing incidence of leprosy in Europe was not caused by the evolution of *M. leprae* to a distinct genotypes that was less virulent or less transmissible. A more probable explanation is that changing social conditions reduced the rate of transmission and that concomitant epidemics of tuberculosis and *Yersinia pestis* (plague) resulted in high rates of mortality in leprosy infected individuals, effectively reducing the reservoir of transmissible infection [17] and hastening the selection of resistant host genotypes in the human population. A further factor may have been an increase in immunological resistance to leprosy in the population associated with exposure to the increasing levels of tuberculosis [8,18].

The genomes of the *M. leprae* from skeletons Sk14 and Sk27 group firmly with other type 2F strains. Moreover, the most closely related *M. leprae* strain in the worldwide phylogeny (Figure 2) to Sk14 is another ancient St Magdalen skeleton, Sk8, described in our previous studies [7,10]. Both individuals were contemporaneous children or adolescents, illustrating vividly the epidemic and conserved nature of leprosy at this site.

Until recently, type 2 *M. leprae* had only been identified in Asia and the Near East. It had been assumed that these type 2 strains gave rise to type 3 strains and moved with human migration to become the archetypal strains in Western Europe [8]. However, we and others have shown the presence of type 2 strains in Europe [10,19] in the 11^th^ and 12^th^ centuries, alongside the more typical type 3K, 3M and 3I strains [8,20] (Table 3). It is now clear that, at least at Winchester, type 2F strains, rather than being unusual, may well have been a co-dominant or even the predominant strain during the 11^th^ century. The lack of type 2F strains and presence of type 3I strains in later burials both here at Winchester, and elsewhere in Europe [21] may be indicative of the shift away from type 2F strains towards the type 3 strains of leprosy typically seen in later European and North American (thought to be epidemiologically related to European strains [7,8]) strains. It is difficult, from these data, to determine whether the type 2F strains identified at St Mary Magdalen were truly endemic to the area, or whether they were a relatively new introduction, possibly associated with trade or pilgrimage, evidence for which is graphically illustrated by the presence of a scallop shell in the grave of Sk27. Whatever their origins, the apparent displacement of type 2F strains by type 3 strains in later archaeological and contemporary samples is unlikely to have been due to a selective pressure as there are very few genetic differences between strains, and those that are present are not predicted to encode phenotypic changes. An alternative model describing a shift towards a type 3 European *M. leprae* population driven by allelic drift associated with a waning European leprosy epidemic would seem more plausible.

**Table 3:** **Details of ancient *M *. *leprae *** **strains with known genotypes, ordered by date**

**Location**	**Case**	**Date (century or cal AD)**	**Type**	**Reference**
Ustyurt plateau, Uzbekistan	5b	1^st^-4^th^ C	3L	[22]
Dakhleh Oasis, Egypt	K2-B116	4^th^-5^th^ C	3K/L/M	[8]
Kiskundorozsma, Hungary	KD271	7^th^ C	3K	[8]
Kovuklukaya, Turkey	KK20/1	8^th^-9^th^ C	3K	[8]
Radasinovic, Croatia	2A	8^th^-9^th^ C	3	[21]
Radasinovic, Croatia	3A	8^th^-9^th^ C	3	[21]
Norwich, UK	11287	10^th^-11^th^ C	3	[21]
Püspökladany, Hungary	222	10^th^ C	3K	[8]
Püspökladany, Hungary	503	11^th^ C	3M	[8]
Norwich, UK	11503	10^th^-11^th^ C	3	[21]
Norwich, UK	11784	10^th^-11^th^ C	3	[21]
Winchester, UK	Sk14	995-1033 AD	2F	[7,10], this study
Winchester, UK	Sk8	1010 -1160 AD	2F	[10]
Winchester, UK	Sk27	1020-1162 AD	2F	this study
Sigtuna, Sweden	3077	1032-1155 AD	2F	[7,19]
Refshale, Denmark	Refshale_16	1046-1163 AD	2F	[7]
Winchester, UK	Sk7	10^th^-12^th^ C	3I	[10]
Winchester, UK	Sk19	10^th^-12^th^ C	3I	[10]
Sigtuna, Sweden	3092	10^th^-14^th^ C	2F	[19]
Sigtuna, Sweden	3093	10^th^-14^th^ C	3I	[19]
Winchester, UK	Sk2	1268-1283 AD	3I	[7,10]
Odense, Denmark	Jorgen_625	1293-1386 AD	3I	[7]
Ipswich, UK	1914	1263-1538 AD	3I variant	[13,21]
Odense, Denmark	G483	1275-1560 AD	3I/J	[21]
Aomori, Japan	sk26	18^th^-19^th^ C	1	[23]

Adapted from Monot *et al*. [8].

## Conclusion

Through the study of bioarchaeology and specifically ancient DNA, we are gaining a greater understanding of the medieval European leprosy epidemic. By concentrating on a single well-characterized site typical of British *leprosaria*, St Mary Magdalen near Winchester, we have gained a detailed and almost personal insight into the wider epidemic and how it changed across the centuries. Our findings reveal that at this site during the 11^th^ century, leprosy strains were typically part of a closely related group of type 2F strains, not the type 3 strains that predominate in later European samples.

## Methods

### Bone retrieval and methods

The skeletons were excavated between 2009 and 2011 by a team from the University of Winchester. The burials were recovered as articulated remains from single graves and associated grave fills were subject to 100% sampling. Pathological conditions were recorded and photographed in detail and diagnosed with reference to appropriate sources [11,12]. All necessary permits were obtained for the described field studies, including a licence (−0070) to exhume and retain human remains, provided by the Ministry of Justice, 102 Petty France, London, SW1H 9AJ.

### Bone sampling and DNA extraction

Samples of bone were taken from the rhino-maxillary region of skeletons Sk14 and Sk27 to maximize the chances of recovering *M. leprae* DNA. Measures were taken to minimize the opportunities for cross-contamination between cases and, use of modern DNA as a positive control was avoided completely [10]. The vomer region of a skeleton, Sk12, with no signs of leprosy was sampled as a negative control and was treated identically to the other samples throughout. The bones were ground to a fine powder and DNA extracted using the NucliSens™ extraction kit (bioMériux Limited, Boxtel, The Netherlands) as described previously [13] in laboratories physically separate from rooms in which leprosy DNA has been previously amplified.

### PCR amplification of specific loci

Real-time PCR assays were carried out on a Mx3005P qPCR system (Agilent Technologies, Wokingham, UK). Methods for the PCR amplification of the multi-copy RLEP locus, the single copy locus coding for the 18 kDa antigen (ML1795), and for the *M. tuberculosis* specific multi-copy loci IS*1081* locus are all described by Taylor, 2013 [10].

### Whole genome amplification, enrichment and analysis

DNA was extracted, enriched and sequenced either at the University of Surrey alone, for Sk27, or independently at both the University of Surrey and University of Tübingen for Sk14. Both protocols are fully described by Schuenemann *et al*. [7]. Both involved removing uracil residues with USER enzyme (New England Biolabs, Hitchin, UK), repairing the DNA, ligating linkers to the ends, and PCR amplifying before enriching for *M. leprae* sequences using microarrays (once at the University of Tübingen and twice at the University of Surrey). Samples were pair-end sequenced with double indices on HiSeq2000 or MiSeq machines.

At the University of Surrey, reads were quality controlled and aligned to the *M leprae* TN genome [9] using Bowtie2 [24]. Duplicate reads were removed with MarkDuplicate script [25] and SNPs assigned. The criteria for inclusion in the SNP/indel list were that polymorphs had to be outside of annotated repeat regions, to have a read depth of more than 5 reads in the sample, but less than 5 reads in the control sample Sk12, have a QUAL score of > =50 (not required for longer indels) and appear in more than 80% of reads. Indels had an additional requirement that all qualifying reads had to span the indel.

At the University of Tübingen the reads were first quality controlled using FastQC, followed by adapter clipping, merging of corresponding paired-end reads and, finally, quality trimming of the resulting reads. The reads were mapped against the *M. leprae* TN genome as a reference using the Burrows-Wheeler Aligner (BWA). Mapped reads were subject to duplicate removal using Samtools’ rmdup method. The Genome Analysis Toolkit (GATK) was then used to generate a mapping assembly and to call SNPs for each strain. The TN reference base was called if the quality score was at least 30 and the position was covered by at least 5 reads. A variant position (SNP) was called if the position was covered by at least 5 reads and the fraction of mapped reads containing the SNP was at least 90%.

To be included in phylogenetic analysis, SNPs had to either be identified at both centers, or identified at only one center, with no contradictory data from the other. Phylogenetic analysis were carried using MEGA5 [26] using Maximum Likelihood (using the Tamura-Nei model), Maximum Parsimony (using Max-mini Branch-&-bound algorithm) and Neighbour-Joining (using the number of differences method) methods.

### Availability of supporting data section

All raw read files have been submitted to the related trace archive of the National Center for Biotechnology Information Sequence Read Archive entry PRJNA200950. Alignments and phylogenetic trees have been submitted to TreeBASE with study ID 15537.

## Competing interests

The authors declare that they have no competing interests.

## Authors’ contributions

TAM planned the study, prepared DNA for NGS sequencing, analyzed the NGS data and drafted the manuscript. VJS prepared DNA for NGS sequencing; SR co-ordinated the archaeology and contributed to the manuscript; GMT planned the study, carried out DNA extractions, carried out and contributed to the manuscript; HW contributed to the analysis of the genome data; PS prepared DNA from the archaeological material, carried out some of the SNP analysis and contributed to the manuscript. KT contributed to the excavations and carried out the osteological examinations; JH contributed to the preparation of the DNA for NGS sequencing; STC contributed to the SNP analysis and the manuscript; AMK contributed to the NGS analysis; KN contributed to the NGS analysis and phylogenetics; JK contributed to the manuscript; GRS was involved in planning the study and contributed to the manuscript. All authors approved and read the final manuscript.

## Supplementary Material

Additional file 1: Table S1 A summary of the known polymorphic loci of *M. leprae* as described by Schuenemann *et al *[7] with additional data for Sk14 and Sk27. Click here for file

Additional file 2: Figure S1 The evolutionary histories of Sk27, Sk14 and the strains described by Schuenemann *et al*, 2013 [7], inferred using Maximum Likelihood, Neighbour Joining and Maximum Parsimony methods. Click here for file
