# Supplementary material for: Mycobacterium leprae genomes from a British medieval leprosy hospital: towards understanding an ancient epidemic
Source: BMC Genomics. 2014 Apr 8;15:270. doi: 10.1186/1471-2164-15-270 (PMC4234520; doi:10.1186/1471-2164-15-270)
Supplement: Additional file 2: Figure S1 — The evolutionary histories of Sk27, Sk14 and the strains described by Schuenemann et al, 2013 [7], inferred using Maximum Likelihood, Neighbour Joining and Maximum Parsimony methods. [file 1471-2164-15-270-S2.docx]

**Supplementary data for**

***Mycobacterium leprae* genomes from a British medieval leprosy hospital – understanding an ancient epidemic.**

Mendum TA^1^, Schuenemann VJ^2^, Roffey S^3^, Taylor GM^1^, Singh P^4^, Tucker K^3^, Wu H^1^, Hinds J^5^, Kierzek AM^1^, Nieselt K^6^, Krause J^5^, and Stewart GR^1^*

Additional file 2

Supplementary Figure 1. Phylogenetic analysis of *M. leprae* genomes.

**a) b)**

**c)**

The evolutionary histories of Sk27, Sk14 and the strains described by Schuenemann *et al*, 2013 [7], inferred using a) Maximum Likelihood, b) Neighbour Joining and c) Maximum Parsimony methods all conducted with MEGA5.2 [23]. The percentage of replicate trees in which the associated taxa clustered together in the bootstrap test (500 replicates) are shown next to the branches. Positions with less than 95% coverage were eliminated. There were a total of 419 informative positions in the final dataset. The tree is drawn to scale, with branch lengths measured in the number of substitutions per site or number of base differences per sequence.
